# Osseodensification vs. Conventional Osteotomy: A Case Series with Cone Beam Computed Tomography

**DOI:** 10.3390/jcm13061568

**Published:** 2024-03-09

**Authors:** José Adriano Costa, José Manuel Mendes, Filomena Salazar, José Júlio Pacheco, Paulo Rompante, Joaquim Ferreira Moreira, José Diogo Mesquita, Nuno Adubeiro, Marco Infante da Câmara

**Affiliations:** 1 UNIPRO—Oral Pathology and Rehabilitation Research Unit, University Institute of Health Sciences (IUCS-CESPU), 4585-116 Gandra, Portugal; jose.costa@iucs.cespu.pt (J.A.C.); filomena.salazar@iucs.cespu.pt (F.S.); julio.pacheco@iucs.cespu.pt (J.J.P.); paulo.rompante@iucs.cespu.pt (P.R.); joaquim.moreira@iucs.cespu.pt (J.F.M.); marco.camara@iucs.cespu.pt (M.I.d.C.); 2 Department of Radiology, School of Health of Porto/Polytechnic Institute of Porto (ESS/IP), 4200-072 Porto, Portugal; jdm@ess.ipp.pt (J.D.M.); nca@eu.ipp.pt (N.A.); 3 EPIUnit, Institute of Public Health, University of Porto, 4200-072 Porto, Portugal; 4 Laboratory for Integrative and Translational Research in Population Health (ITR), 4050-600 Porto, Portugal

**Keywords:** osteotomy, cone beam computed tomography, dental implant, bone density

## Abstract

**Introduction**: Osseodensification is a non-extraction technique using specially designed drills to increase bone density while extending an osteotomy, allowing bone to be preserved and condensed by compacting autograft during osteotomy preparation, increasing bone density around implants, and improving mechanical stability. **Aim**: The objective of this study is to compare conventional osteotomy and osseodensification protocols in implant placement and analyze whether there are differences in bone density. **Materials and Methods:** Study variables were defined, namely, osseodensification technique, conventional osteotomy technique, bone density, sex, area of location, implant dimensions, implant dimensions, and implant stability. Eligibility and exclusion criteria were defined. A step-by-step surgical protocol was developed. The surgeon and radiologist underwent intra-examiner calibration. A total of 15 patients were selected according to the eligibility criteria, and a total of 41 implants were inserted, 20 implants by conventional osteotomy and 21 by osseodensification. A cone beam computed tomography was performed one year after prosthetic rehabilitation to estimate bone density. Data were collected and recorded, and in the analysis of the association of variables, non-parametric tests were applied. **Results:** Significant statistical results were found in bone density values, with higher values being obtained with the osseodensification technique, that is, median density values of 1020, and median density values of 732 for the bone drilling technique. The results of the correlation between bone density in both techniques and sex, primary implant stability, implant dimensions and location area were statistically non-significant. **Conclusions:** Within the limitations of this study, there are differences in bone density between conventional osteotomy and osseodensification protocols. Bone density is increased with osseodensification over a study period of one year.

## 1. Introduction

Dental implants are a reliable therapy option to replace missing teeth and to restore the esthetics, chewing function, and communication of patients [1], yielding high treatment success rates [2]. This success depends on the osseointegration process. The stability of the implant is an indirect indication of the osseointegration and is observed at two levels, the primary stability and the secondary stability. Primary stability is the mechanical stability between the implant and bone, obtained through mechanical friction between the implant surface and bone walls of the osteotomy site. Secondary stability allows the establishment of biological stability resulting from the formation of new bone cells on the implant surface. Adequate primary stability results in the establishment of an appropriate secondary stability. Primary stability can be measured at the time of implant placement using insertion torque. An important correlation is noted between bone density and insertion torque, both of which are determinants of implant stability. A higher host bone density translates into a greater insertion torque and increased implant stability. There are other noninvasive methods to evaluate implant stability, including Periotest^®^ (Bensheim, Germany) and resonance frequency analysis (RFA) [3]. Although for the evaluation of the primary stability, it is sufficient to measure the value of the implant insertion torque, since this parameter is easily accessible [4]. Insertion torque depends largely on the characteristics of the implant design, the density of the bone, and the osteotomy technique [5].

Bone density depends on factors such as sex, with higher bone mass reported in males [6]. Bone mass is proportional to the modulus of elasticity of the bone. Higher bone density translates into a higher elastic modulus, resulting in a larger area in contact with the implant [7].

Different areas of the maxilla and mandible have different densities. Using computed tomography (CT) and cone beam computed tomography (CBCT), the following sequence, in descending order, of bone density in various areas was reported, namely, anterior mandible, anterior maxilla, posterior mandible, and posterior maxilla [8].

There is no reported consensus on the effect of implant dimensions, width and length, in different bone types [3], although it has been described that larger implant sizes lead to higher insertion torques in soft bone and that the increase in diameter seems to play a greater role than the implant length, so wider implants are preferable in cases of soft bone [9].

There are several techniques that can be used to increase the bone density and primary stability, such as under-preparation of the implant bed. However, the host-to-implant early response can be affected because a high degrees of bone mechanical strain can evolve after implant placement [10]. A variation of the under-preparation technique is the stepped osteotomy, which can be a viable method to increase primary stability [11]. In 1994, Summers described the use of osteotomes to condense bone [12]. Still, this technique can cause fracture or bone displacement and vertigo [13]. Another reported technique to expand bone and create an osteotomy without removing but rather displacing bone is ridge expansion. However, a fracture of the buccal plate during this procedure can affect the stability of the implant insertion [14].

The concept of osseodensification was developed by Huwais in 2013 [15]. It introduced a new method of biomechanical bone preparation performed for dental implant placement with specially designed densifying burs called Densah^®^ burs (Versah^®^ LLC, MI, USA). In the conventional drilling technique, the drills cut and remove the bone tissue, but when using Densah^®^ burs, the drill design allows the creation of an environment that increases the initial primary stability through densification of the osteotomy site walls utilizing non-subtractive drilling. The Densah^®^ burs have a special design with many lands with a large negative tilt angle, which work as noncutting edges to increase the density of the bone as they expand an osteotomy. These densifying burs have four or more lands and flutes that smoothly compact the bone. Densifying burs are surgical devices with a cutting chisel edge and a tapered shank. Hence, as they enter deeper into the osteotomy, they have a progressively increasing diameter that controls the expansion process. These burs are used with a standard implant surgical unit. They can densify bone by rotating in the noncutting direction (counterclockwise at 800–1200 rpm) or drill bone by rotating in the cutting direction (clockwise at 800–1200 rpm) [16].

In 1998, Mozzo et al. introduced cone beam imaging technology, which, depending on the specific configurations of the cone beam computerized tomography (CBCT) unit, allows three-dimensional images to be obtained with relatively low radiation doses [17]. Although CBCT has made a speedy ingress into the field of dentistry, it is not devoid of downsides, as the image’s clarity is affected by artifacts, noise, and poor soft tissue contrast [18]. Artifacts are the main limitation of CBCT, that is, image flaws unrelated to the digitized object caused by metals such as dental implants [19]. Image noise is caused by a large volume being irradiated during CBCT scanning, causing heavy interactions with tissues resulting in scattered radiation, which in turn leads to nonlinear attenuation by the detectors. Regarding poor soft tissue contrast, there are factors that restrict the contrast resolution of CBCT, such as enhanced image noise, embedded flat-panel detector-based artifacts, and the divergence of the X-ray beam. These downsides may be related to the cone beam projection geometry, detector sensitivity, and contrast resolution [20]. However, these described aspects cannot be generalized, as different CBCT manufacturers present different spatial resolutions (voxel sizes), fields of view (FOVs), patient positioning systems, or scanning durations, which will influence the quality and interpretability of the scans [21]. More specifically, CBCT is capable of providing voxel sizes from 50 μm up to 600 μm; the smaller the voxel size, the higher the spatial resolution. The FOVs in CBCT vary from being suitable for a single jaw or a few teeth to full craniofacial imaging. Most of the machines use either one or a few prefixed FOVs (up to 23 different FOVs) according to different indications, although, to some extent, they can be adjustable. Regarding patient position, standing position systems are frequently used, although seated and supine position devices may allow a reduction in patient motion-related artifacts. In CBCT technology, the single rotation allows a rapid scan time ranging from 2 to 45 s, depending, for example, on the acquired number of projections and rotation arc of the scan [22].

Additionally, the improvement in reconstruction algorithms and newer metal artifact reduction algorithms is becoming increasingly common [23].

Although many studies on osseodensification drilling have been reported [16,24,25,26,27,28,29], few studies have compared conventional drilling techniques in bone density with CBCT [30,31].

This study aimed to compare conventional drilling and osseodensification protocols in implant placement and analyze whether there are differences in bone density.

## 2. Materials and Methods

### 2.1. Study Design

The study followed the protocols of the Declaration of Helsinki and was approved by the IUCS-CESPU ethical committee in February of 2019 with the serial number 04/CE-IUCS/2019. Informed and detailed consent was revised and signed by each subject, and the investigation was conducted between March 2019 and July 2023.

The present study was designed as a case series, with a one-year follow-up in a convenience sample of 15 patients, partially edentulous, who attended the oral implantology post-graduation degree of the IUCS-CESPU, Portugal, with the purpose of oral rehabilitation with dental implants.

Study variables were defined, namely, osseodensification technique, conventional osteotomy technique, bone density, sex, area of location, implant dimensions, and implant stability. Eligibility and exclusion criteria were defined. The inclusion criteria included two steps. The first inclusion criteria stages were as follows: adult patients over 18 years old, both sexes, good general health, no chronic systemic diseases such as osteoporosis, diabetes mellitus, heart diseases, as such conditions may complicate the surgical procedure or the healing process, bilateral and/or adjacent presence of the same edentulous area, and characteristics in the maxilla or mandible for more than 6 months requiring placement of at least one dental implant, good oral hygiene, and motivation and willingness to participate. All patients who signed a consent form and only patients with successful dental implant placement using both techniques and successful prosthetic crown rehabilitation 1 year prior and regular follow-up visits to assess oral hygiene were eligible.

In the second inclusion criteria step, all patients selected with eligibility criteria in the first step were submitted to radiographic evaluation, panoramic radiography, and CBCT to evaluate jaws, dentition, proximity to vital structures, estimation of bone volume, implants selection, implants dimensions, and surgical technique.

The exclusion criteria were as follows: presence of any chronic systemic disease such as osteoporosis, diabetes mellitus, and heart disease, as such conditions may complicate the surgical procedure or the healing process, smoking, alcohol or drug abuse, previous or ongoing bisphosphonate therapy, any soft or hard tissue pathology, bruxism, patients with a medical condition impeditive of CBCT scan, patients submitted to bone graft, presence of CBCT artifacts, and implants placed adjacent to corticalized bone or anatomical structures.

Of the 15 patients, according to the eligibility criteria, 8 were male and 7 were female, aged in the range of 34 to 64 years old.

An initial radiographic study, orthopantomography, and CBCT were performed on selected patients. The location of implants was selected in each patient. The implants and their dimensions were selected individually by location. The surgical technique was selected for each of the implants. A total of 41 implants were placed, 20 by the conventional osteotomy technique and 21 by the osseodensification technique.

### 2.2. Surgical Step

All surgical procedures were performed by an experienced oral surgery specialist submitted to intra-examiner calibration at the medicine and oral surgery department of the IUCS-CESPU.

According to Misch’s protocol [32], 48 h prior to surgery, an antibiotic regimen was prescribed. After patient preparation, local anesthesia was administered (articaine hydrochloride 4% with adrenaline 1:100,000 (Artinibsa by Inibsa^®^, Barcelona, Spain)).

The implants were placed in the adjacent and/or contralateral area, according to the study design and eligibility criteria. On one area, the drilling protocol was performed with conventional osteotomy and, in the other, with the osseodensification technique.

The 20 implants placed by the conventional osteotomy technique followed the standard drilling protocol for BLT SLA Straumann^®^ implants, where the osteotomy was performed using the pilot drill, namely, 1200 rpm with copious irrigation. Conventional drills were used sequentially per the diameter protocol, and the subsequential drills were reduced to 800 rpm and 400 rpm.

The 21 implants placed by osseodensification on the bone crest were performed with a crestal incision on the edentulous area, and a full thickness mucoperiosteal flap was raised. The extension of the flap was dependent on the case demand for surgical accessibility and visualization. Following flap elevation, one implant site was prepared using the osseodensification protocol with Densah^®^ burs (Versah^®^ LLC, MI, USA), where osteotomy preparation was performed using the pilot drill (clockwise drill speed 1200 rpm with copious irrigation), with the correct position and alignment assessment through parallel pins. Afterward, depending on the implant diameter selected, site preparation proceeded in densification mode through the sequential stepped drilling (counterclockwise drill speed of 1200 rpm with copious irrigation) using gradually wider diameter burs according to the Densah^®^ protocol (Table 1).

In both techniques, BLT SLA Straumann^®^ (Straumann® Group, Villeret, Switzerland) implants were installed with a W&H^®^ unit (W&H, Austria) set at 30 rpm with torque of 30 N/cm. During implant insertion, the maximum insertion torque value was recorded in the same implant unit, starting at 20 N/cm. The placement torque was increased in steps of 5 N/cm, from stopping frictional rotation before complete insertion of the implant until reaching 35 N/cm. A manual ratchet was used to place the implants at the desired depth when the insertion torque was greater than 35 N/cm. After placing the implants, healing screws were installed with a tightening of 10 N/cm on the implants and the flaps were repositioned and sutured with 5–0 polyamide. (Resolon^®^ by Resorba, Nurnberg, Germany).

All data were recorded by surgical technique, location, implant dimensions, and insertion torque in each implant.

All patients received immediate postoperative instructions. The sutures were removed after 7 days. The oral hygiene measures were reinforced, and the stability and osseointegration of the implants were monitored.

### 2.3. Reestablishment of Function Step—5–7 Months Later

Five months later, patients were scheduled, and an appropriate healing abutment was inserted considering the emergence profile and gingival height.

Seven months later, scans with Trios 3 (3Shape^®^, Copenhagen, Denmark) were taken to finalize the zirconia prosthetic crown. Rehabilitation was completed with the placement of zirconia crowns.

Data were recorded by surgical technique, location, and implant in each patient.

### 2.4. Data Extration—One Year Later

The radiographic examination and the CBCT’s diagnosis to estimate the bone density were performed by a single blinded specialist in radiology who had previously carried out intra-examiner calibration at the radiology service at the Higher School of Health, Polytechnic Institute of Porto (ESS/IPP), Portugal. The CBCT unit Newtom^®^ GO Ref. 70BE 3D (Cefla S.C., Imola, Italy) settings were standardized, and the tube was set to 90 kVp, 6 mAs, and an exposure time of 5.6 s. The plane of the jaw was used as a reference for beam orientation.

**Figure 1:**
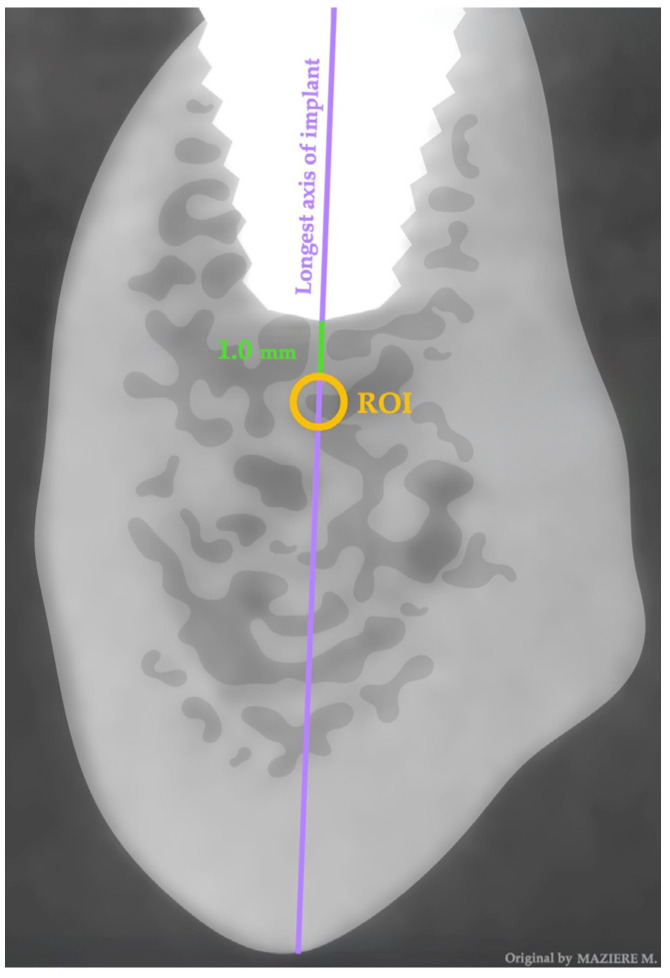
CBCT section showing radiographic evaluation methodology.

The data extraction was processed, and the values quantified by Horos TM^®^ software 3.3.6 (Horos Project, Brooklyn, NY, USA). The acquisition volume was processed while respecting the orthogonal axes of the implants, reconstructing images 1 mm thick and 1 mm spacing sagittally to the longest axis of the implant, obtaining V-L view images of the implant to be studied. We applied a region of interest (ROI) dimension of 1 mm for the entire cancellous bone within the same view 1 mm apical to the longest axis of the implants placed through osseodensification and the conventional technique while taking into consideration an implant apex distance of at least 2 mm for the mandibular canal, mental foramen, nasal cavity, and floor and wall of the maxillary sinus (Figure 1). This ROI selection was undertaken taking into consideration that the smaller the ROI, the higher the spatial resolution, and to avoid proximity with corticalized bone. The bone mineral density was analyzed within the internal area of the ROI and expressed in density value (DV).

### 2.5. Data Analysis

All data were analyzed by an independent statistician using SPSS statistics software 29.0 (IBM^®^ Corporation; Armonk, NY, USA), and the significance was set at a *p*-value < 0.05.

For analysis purposes, it was decided to distribute the variables by implant, since each individual has at least one implant from each of the techniques. Therefore, the description of the sample in terms of number of implants assumes different values from the population sample. The extracted data did not follow a normal distribution, as they were obtained from a convenience sample, which is why non-parametric tests were carried out to study the data, namely, Mann–Whitney, Kruskal–Wallis, and Spearman. Convenience samples, with all their recognized limitations, continue to be very useful tools in health research. The representativeness of the sample is according to Cochran’s [33] with a 95% confidence interval.

Statistical inference tests were applied to evaluate differences between sex and the respective bone density of the two techniques used using the Mann–Whitney test. The Kruskal–Wallis test was used to assess differences in bone density with both techniques, according to the different locations. The correlation between bone density with both techniques, implant dimensions, and implant stability was also assessed using the Spearman test.

## 3. Results

### 3.1. Sample Characterization by Implant Distribution According to Sex and Surgical Technique

The implant’s distribution according to sex and surgical technique is presented in Table 2.

### 3.2. Sample Characterization by Maxillary and Mandibular Implant’s Location

The implant’s distribution according to maxillary and mandibular implant’s location is presented in Table 3.

### 3.3. Sample Characterization by Maxillary and Mandibular Implant’s Location and Surgical Technique

The implant’s distribution according to maxillary and mandibular implant’s location and surgical technique is presented in Table 4.

### 3.4. Sample Characterization by Implant’s Dimensions According Surgical Technique

The implant’s dimensions according to surgical technique are presented in Table 5.

### 3.5. Primary Stability According to Surgical Technique

The implant’s insertion torque according to surgical technique is presented in Table 6.

### 3.6. Correlation between Bone Density in Both Techniques with Area of Location Implant Stability, Implant Dimensions, and Sex

The correlation between bone density in osseodensification and the conventional osteotomy technique with implant stability (insertion torque) and implant dimensions (width, and length), areas of location, and sex is presented in Table 7 using the non-parametric Spearman test.

### 3.7. Bone Density According to Surgical Technique

Bone density according to surgical technique is presented in Table 8.

## 4. Discussion

There are several radiologic methods used to measure bone density. DXA is considered the gold standard for bone density analysis. However, DXA does not fully capture the three-dimensional (3D) bone microstructure and morphology as the information is obtained two-dimensionally (2D) [34]. Micro-CT has a high accuracy in micrometers for trabecular parameters and is considered the “gold standard” for bone morphology and micro-structure, such as bone volume and bone fraction, but is limited to the ex vivo bone model [35]. Computed tomography (CT) is a well-established method able to provide a larger field of view, superior signal and contrast-to-noise ratios, and more accurate Hounsfield unit values when compared to CBCT [36]. CBCT has advantages over conventional CT and DXA as it is more affordable, emits a lower radiation dosage, has faster acquisition, and yields higher image quality, but variations found are associated with the use of different CBCT units, different voxel values, different imaging parameters, and different positioning sites, and by measuring different bone regions [37].

However, it is important to analyze whether the doubts about the limitations of CBCT for assessing maxillary bone mineral density are real [22] or whether the limitations should simply be interpreted as a limitation of the study or whether it will be worth investing in improving the methodology’s reproducibility and explore the analysis of the result’s reproducibility.

Animal study models indicate that osseodensification increases bone density, primary stability, and the percentage of bone–implant contact. Furthermore, they state that by preserving bone mass, the healing process is accelerated due to the bone matrix, cells, and biochemicals maintained and self-grafted along the implant bed and at the apex of the osteotomy through compaction of the cancellous bone [16].

Animal study models report that implants placed in low-density bone using the osseodensification surgical technique presented significantly higher insertion torque values than with conventional osteotomy, namely 65 N/cm and 35 N/cm, regardless of the implant area [24,25].

Furthermore, animal study models that compared the surgical techniques of conventional osteotomy and osseodensification confirm these facts through histomorphological analysis [25,26,27] and computed tomography (CT) and demonstrated the presence of autogenous bone fragments in the osseodensification location, mainly in bone with low mineral density, compared to conventional drilling [28].

The split-mouth clinical studies, an in vivo model, to compare classical osteotomy with osseodensification are, to date, apparently the most consensual. Their study designs differ methodologically from each other in sample size, variables under study, implant location, measuring variables method, and study follow-up.

This kind of study reported that when using CBCT to measure bone crest levels and implant stability using RFA, where only 20 implants were placed in the anterior region of the maxilla, demonstrated no statistically significant results [29].

Aloorker et al. [30], in the same clinical study design, inserted 20 implants bilaterally in the posterior maxillary region of 10 patients and compared the effects of osseodensification and conventional osteotomy, in terms of crestal bone level and radiographic bone density assessed at different time intervals using CBCT. They found no statistical differences between the two techniques in terms of crestal bone level, although they reported an increase in radiographic bone density adjacent to the implants that remained relatively dense over a period of 6 months [30]. Another split-mouth clinical study was performed by Hassan et al. [31] inserting 14 implants in 7 female patients through osseodensification and conventional osteotomy. They analyzed bone density with a CBCT performed immediately post-operatively, at 7 months, and at 12 months. Despite finding statistical differences in bone density in the immediate post-operative radiographic examination, they reported no statistically significant differences at 7 and 12 months [31]. The last timeframe is equal in our study and is contrary to our results.

Recently, the scientific literature has attributed to drills specially designed for the osseodensification technique the increase in bone density as they expand an osteotomy, the increase in bone preservation, bone condensation through the compacted autograft during osteotomy preparation, and the increase in bone density around the implants, as no bone is extracted, thus improving the mechanical stability of the implant [38].

In our study, most implants were placed in male patients, 53.3%, which is contrary to most studies, as there is a greater predisposition among females to resort to implant treatment [39]. Regardless of the groups and regardless of the surgical technique, most implants were placed with an insertion torque greater than 35 N/cm (Table 6); however, the result of the association between the variable primary stability and bone density with both techniques was not statistically significant (Table 7).

In the analysis of the association between sex and the bone density, regardless of the surgical technique, namely, osseodensification and conventional osteotomy, no statistically significant results were found (Table 7).

The result of the association between the variable bone density with surgical technique and implant location was also not statistically significant (Table 7), but when we restricted ourselves to the analysis of the variable bone density and osseodensification, the results were statistically significant (*p* = 0.028), with higher values being obtained with the osseodensification, median 1020 DV, when compared to the conventional osteotomy, the drilling technique, median 732 DV, over a period of one year after implant placement, supporting the use of the osseodensification technique to increase bone density (Table 8).

The posterior maxillary location was the most used location, 56.1%, followed by the posterior mandibular region, 22.0%. Regardless of the eligibility criteria and the implant’s location selection criteria, and although there were no statistically significant results of density with both techniques between locations (Table 7), the identification of increased density in these locations can be conditioned by the lower characteristic density of these edentulous posterior zones [40].

The most commonly used were 4.1 (diameter) and 10 mm (length), and the result of the association between the variable implant’s dimensions and density with osseodensification and the conventional osteotomy technique was that it was not statistically significant (Table 7).

All the results obtained by these studies should be analyzed and observed with caution, since the studies have several limitations and risks of bias inherent to this kind of study, such as the limited sample size, patient demographics, and methodology. These limitations apply to the present study.

Future research must be improved to parameterize methodology and data analysis.

## 5. Conclusions

Within the limitations of this study, there are differences in bone density between conventional osteotomy and the osseodensification protocols.

Bone density is increased with osseodensification over a study period of one year.

## Figures and Tables

**Table 1:** Decision tree for osseodensification protocol.

	Soft Bone					Hard Bone (Mandible)				
Implant Diameter	Drill	Bur 1	Bur 2	Bur 3	Bur 4	Drill	Bur 1	Bur 2	Bur 3	Bur 4
3.3	Pilot Drill	VT1828	-	-	-	Pilot Drill	VT1828	VS2228	-	-
4.1	Pilot Drill	VT1525	VT2535	-	-	Pilot Drill	VT1828	VT2535	VT2838	-

**Table 2:** Implant distribution according to sex and surgical technique using the Mann–Whitney non-parametric test.

Technique	Sex	Frequency (*n*)	Percentage (%)
	Male	12	57.1
Osseodensification	Female	9	42.9
	Total	21	100
	Male	10	50
Conventional	Female	10	50
	Total	20	100

**Table 3:** Maxillary and mandibular implant’s location.

Jaw	Location	Frequency (*n*)	Percentage (%)
Maxilla	Anterior	7	17.1
	Posterior	23	56.1
Mandible	Anterior	2	4.9
	Posterior	9	22.0
Total		41	100

**Table 4:** Implant location distribution according to technique.

Technique	Location	Frequency (*n*)	Percentage (%)
Osseodensification	Anterior Maxilla	3	14.3
	Posterior Maxilla	12	57.1
	Anterior Mandible	1	4.8
	Posterior Mandible	5	23.8
	Total	21	100
Conventional	Anterior Maxilla	4	20.0
	Posterior Maxilla	11	55.0
	Anterior Mandible	1	5.0
	Posterior Mandible	4	20.0
	Total	20	100

**Table 5:** Implant’s dimensions according to surgical technique.

Implant Dimensions	Implant Dimensions (mm)	Osseodensification (*n*)	Conventional (*n*)	Total (*n*)	Total (%)
Width	3.3	7	5	12	29.3
	4.1	14	15	29	70.7
				41	100
Length	8	4	4	8	19.5
	10	7	10	17	41.4
	12	7	5	12	29.3
	14	3	1	4	9.8
				41	100

**Table 6:** Implant insertion torque according to surgical technique.

Torque Insertion (N/cm)	Osteotomy (*n*)	Osseodensification (*n*)	Total (*n*)	Total (%)
35	6	5	11	26.8
˃35	14	16	30	73.2
			41	100

**Table 7:** Correlation between bone density in both techniques with implant stability, implant dimensions, areas of location, and sex.

Correlation Variables	Insertion Torque (*p*-Value)	Implant Dimension Width (*p*-Value)	Implant Dimension Length (*p*-Value)	Areas of Location (*p*-Value)	Sex (*p*-Value)
Bone density with Osseodensification	0.892 *	0.474 *	0.098 *	0.749 **	0.644 ***
Bone density with Conventional Osteotomy	0.995 *	0.585 *	0.662 *	0.918 **	0.290 ***

* Spearman non-parametric test. ** Kruskal–Wallis non-parametric test. *** Mann–Whitney non-parametric test.

**Table 8:** Bone density values according to technique.

Technique	Parameters	Density Values	*p*-Value
Osseodensification	Median	1020	0.028 *
	IQR	396.5	
	Q1	781.5	
	Q3	1178	
Conventional	Median	732	
	IQR	393.5	
	Q1	615.3	
	Q3	1008.8	

* Mann–Whitney non-parametric test.

## Data Availability

The data can be accessed by contacting the corresponding author.
